# Channel Selectivity of Satellite Transponders with the Antenna Combined with a Size-Reduced Metallic Waveguide Bandpass Filter Having Thin Metamaterial Resonators

**DOI:** 10.3390/s23041948

**Published:** 2023-02-09

**Authors:** Junghyun Cho, Yejune Seo, Wonjae Shin, Eungdon Lee, Sungtek Kahng

**Affiliations:** 1 Department of Information & Telecommunication Engineering, Incheon National University, Incheon 22012, Republic of Korea; 2 The Public Safety AI Research Section, Electronics and Telecommunications Research Institute, Daejeon 34129, Republic of Korea

**Keywords:** waveguide, bandpass filter, metamaterial, equivalent circuit, metamaterial structure, zeroth-order resonance, antenna, channel selectivity

## Abstract

Global and intercontinental networking relies on satellite communication. Its wireless communication system always has antennas and their feed assembly comprising waveguides. This makes the satellite payload heavy and costly. In this paper, a novel method is proposed to effectively reduce the size of a waveguide bandpass filter (BPF). Because the metallic cavities make the conventional waveguide end up with a large geometry, especially for high-order BPFs, very compact waveguide-type resonators having metamaterial zeroth-order resonance (WG-ZOR) are designed on the cross-section of the waveguide and substituted for the cavities. While the cavities are half-wavelength resonators, the WG-ZOR is shorter than one eighth of a wavelength. A substantial reduction in size and weight of the waveguide filter is observed as the resonators are cascaded in series through coupling elements in the X-band much longer than K- or Ka-band. An X-band of 7.25~7.75 GHz is chosen to verify the method as the passband with attenuation of 40 dB at 7.00 GHz and 8.00 GHz as the roll-off in the stopband. The BPF is manufactured using the CNC milling technique. The design is carried out with geometrical parameters, not of the level of 10 μm, but the level of 100 μm, which is good for manufacturers but a big challenge for component designers. The measurement of the manufactured metal waveguide filter reveals that the passband has about ≤1 dB and ≤−15 dB as insertion loss and reflection coefficient and the stopband has ≤−40 dB as attenuation, which are in good agreement with the results of the circuit and simulation. The proposed filter has a length of 3.5 $\lambda_{g}$ as the eighth-order BPF, but the conventional waveguide is 5 $\lambda_{g}$ as the seventh-order BPF for the same area of the cross-section. This metamaterial BPF is combined with a horn antenna. The filter enables the wide-band antenna to distinguish the band of transmission from that of noise suppression. This channel selectivity is obviously observed by the filter integrated antenna test.

## 1. Introduction

Artificial satellites are watching the earth for global-scale wireless networking and scientific data gathering. With telecommunication and weather watches, satellites are indispensable to surveillance and reconnaissance on target regions. Nowadays, LEO satellites are produced for commercial services and deployed in the orbits 800 km to 1000 km above the Earth’s surface; this is sensational for the new concepts such as a short cycle of production, and forming a space-borne network of an overwhelming number of satellites called a constellation [1,2]. Transponders are essential to satellites of any kind and any missions for ground-to-space and satellite-to-satellite wireless links.

The transponder is a wireless communication system that receives RF signals and transmits them whether it is a geostationary orbit (GEO) or LEO satellite. Its configuration is represented by the system block diagram comprising plenty of circuits and components from the receiver to the transmitter. The chain of the signal flow meets the components of signal amplification, channel selection, channel splitting and combining, switching, demodulation and modulation and so forth. For successful tasking, system integration (SI) is important, but the functions of the individual components are more prioritized. Active components connected to control blocks have an increased degree of freedom in meeting the requirements even after SI. As for passive components such as input and output multiplexers (MUXes) for channel splitting and combining, and filters for channel selection, functions are not controllable after fabrication and assembly. Great care should be taken of their design and fabrication. While active components such as MMIC amplifiers do not weigh much, passive components of waveguide (WG) filters and WG MUXes tend to be heavy. Along with the feed horn and reflector antenna, WG passive elements are of great concern in terms of size control and weight control to build a satellite transponder. This becomes a critical matter to the cost required in the making and maintaining of LEO and micro satellites.

In the satellite communication, high-frequency signals must be so strong that they can travel hundreds to thousands of kilometers when emanated from the antenna. The filters, like other passive components in the feed assembly of the antenna, are made out of metallic waveguides to ensure high Q-factors and endure high power and the heat [3,4,5,6,7]. This is why the structures are bulky and heavy. Size-reduction of them is a crucial factor to cost-saving in developing and launching satellites. Sarun and Somsak showed second- and third-order rectangular WG BPFs [3]. Typical sized cavities are combined in series through irises for channel selectivity. Angel and Vincente took an action to reduce the size of the WG filter by integrating coaxial lines with rectangular cavities [4]. The length of their third-order filter is the same as that of the conventional third-order BPFs. Joaquin and Santiago put stepped impedance resonator (SIR) parts with cavity resonators coupled through a mixture of E-plane and H-plane steps [5]. Their WG BPF is longer than four wavelengths. Fernando and Jon meandered the straight topology of a BPF to reduce the area the filter occupies [6]. The mitered E-plane bend-added waveguide sections couple half-wavelength cavities and the total length has almost no change. Valencia and Marco changed a straight geometry to a staircase by opening the two spots on the broad wall of each cavity for coupling [7]. The trace in the longitudinal axis is decreased, but the total length is similar to reference 5. Looking over the latest rectangular waveguide bandpass filters including the reports above, as they follow the design technique of half-wavelength cavities, effective size reduction is nowhere to be found. One might suggest application of metamaterial filter designs seen from microstrip- and CPW-lines like in [8,9] to waveguide components, but this turns out to be not possible because of differences in geometries and modes. Motivation arises for setting up a metamaterial resonator which is much shorter than the typical cavity and geometrically appropriate for the waveguide.

This paper proposes a novel design method of realizing the compact WG BPF of a high order for satellite communication system devising and using the waveguide metamaterial resonator. First, the zeroth-order resonator is formed on the cross-section of the standard waveguide, compliant with the composite right/left-handed (CRLH) circuit model. The geometrical parameters are of unit of 100 μm to avoid the problems of fabrication tolerance, whose lower precision makes this design much tougher than others using the unit of 10 μm with more expensive facilities. Second, the ZOR as a building block for the equivalent network of the eighth-order BPF is substituted for conventional cavities. Third, the resonators are cascaded through short waveguide sections as coupling elements to generate the passband, and the stopbands have high attenuation. Fourth, the WG BPF of ZORs and coupling elements is physically prototyped by the CNC milling technique, which is driving the industry to transform into 3D printing as in [10,11,12]. Fifth, the metallic WG filter is adopted to the feeding component of the horn antenna in the wireless communication system to attain channel selectivity. Sixth, the performances of the individual components and the filter-integrated antenna are investigated by way of real experiments. The suggested method is evaluated by circuit modeling, full-wave EM simulation and measurement. Good agreement between the theoretical and measured results is unveiled from the procedural steps. As noted in the required specifications on the WG BPF, the passband has about ≤1 dB and ≤−15 dB as insertion loss and reflection coefficient, the stopband has 40 dB of noise suppression. Regarding size reduction, the proposed PBF of the metallic waveguide is 3.5 $\lambda_{g}$ for the eighth-order filtering, much smaller than the conventional waveguide filter of the same order. This effect will be obvious for much higher-order filters and MUXes.

## 2. Circuit Modeling of the BPF and the WG ZOR, and Its Geometry

### 2.1. Required Specifications of the BPF for Satellite Wireless Communication

A bandpass filter is needed in the transponder as the communication system for the satellites with the following assessment items and values.

The mathematical approach discovers the function of Chebyshev-type with *N*_order_ = 8 is quite close to the specifications in Table 1 for the amplitude of the bandpass filter as in [13]. It is expressed with the circuit network.

(1) $$t\left(s\right)=\frac{5.356\times 10^{8}s-8.871\times 10^{19}}{s^{2}+1.158\times 10^{9}s+2.193\times 10^{21}}+\frac{5.896\times 10^{8}s+9.092\times 10^{19}}{s^{2}+1.172\times 10^{9}s+2.248\times 10^{21}}\;+\frac{-1.08\times 10^{9}s+4.22\times 10^{19}}{s^{2}+8.982\times 10^{8}s+2.138\times 10^{21}}+\frac{-1.14\times 10^{9}s-4.552\times 10^{19}}{s^{2}+9.329\times 10^{8}s+2.306\times 10^{21}}\;+\frac{7.573\times 10^{8}s-5.793\times 10^{18}}{s^{2}+5.807\times 10^{8}s+2.095\times 10^{21}}+\frac{8.043\times 10^{8}s-6.506\times 10^{18}}{s^{2}+6.153\times 10^{8}s+2.353\times 10^{21}}\;+\frac{-2.253\times 10^{8}s-3.449\times 10^{18}}{s^{2}+2.015\times 10^{8}s+2.073\times 10^{21}}+\frac{-2.41\times 10^{8}s+3.958\times 10^{18}}{s^{2}+2.159\times 10^{8}s+2.379\times 10^{21}}$$

**Figure 1:**
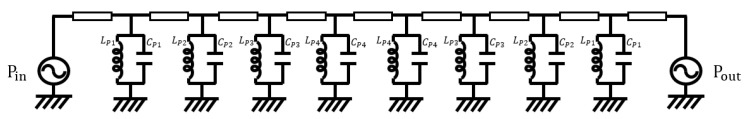
Equivalent circuit network of the bandpass filter.

Figure 1 has eight parallel LC resonators cascaded through transmission-line segments as coupling elements. Equation (1) is the transfer function of the curve fitting the amplitude. The long expression is decomposed into a few terms. The maximum order of the denominator of *t*(*s*) is the same as the number of the resonators. As is done for filter designs, the transmission-line segments or waveguide sections of nearly quarter-wavelength work as an inductor, as mentioned in [14], and put between the resonators for the bandwidth. In the PCB filters and waveguide filters, the LC resonators seen in Figure 1 are changed to the distributed elements, the lengths of which are half-wavelength and odd multiple half-wavelengths. This is the conventional rule which is mostly used owing to simplicity and convenience. If, and only if, this rule is kept, the bandpass filter ends up with a long structure when a higher-order filtering is required, like the specifications in Table 1. The configuration of Figure 1 with the parallel LC resonators leading to conventional resonators is modified into something with new resonators as follows.

**Figure 2:**
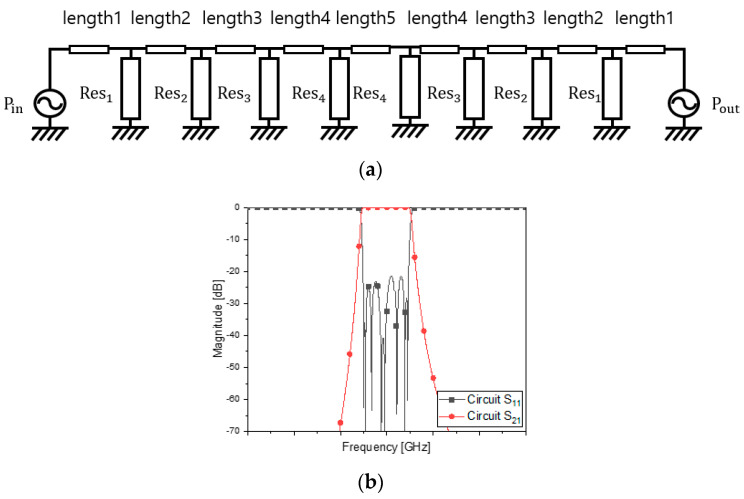
Modified equivalent circuit and its frequency response. (**a**) Circuit. (**b**) S_11_ and S_21_.

The resonators of Figure 2a will be filled with new ones. By satisfying the requirement, circuit calculation gives the unknowns of Figure 1 and Figure 2 the values in Table 2. Figure 2b presents S_11_ as the reflection coefficient and S_21_ as the transmission coefficient of the device. This ideal circuit results in the satisfactory frequency response. The circuit calculation is operated based on the following mathematical procedures.

(2) $$\begin{array}{c} \left[ABCD_{Tot.}^{Circ.}\right]=\left[ABCD_{Res.1}^{Circ.}\right]\left[ABCD_{m_{12}}^{Circ.}\right]\left[ABCD_{Res.2}^{Circ.}\right]\cdots \left[ABCD_{m_{78}}^{Circ.}\right]\left[ABCD_{Res.8}^{Circ.}\right]\\ \left[ABCD_{Res.1}^{Circ.}\right]=\left[ABCD_{L1}^{Circ.}\right]\left[ABCD_{C1}^{Circ.}\right]=\left[\begin{array}{cc} 1 & 0\\ 1/jwL1 & 1 \end{array}\right]\left[\begin{array}{cc} 1 & 0\\ jwC1 & 1 \end{array}\right]\\ \left[ABCD_{m12}^{Circ.}\right]=\left[\begin{array}{cc} cos\beta l & jZ_{0}sin\beta l\\ jY_{0}sin\beta l & cos\beta l \end{array}\right],\;where\;l=length2\\ \left[ABCD_{Tot.}^{Circ.}\right]=\left\{\overset{N-1}{\underset{p=1}{\prod }}\left[ABCD_{Res.p}^{Circ.}\right]\left[ABCD_{m_{p,p+1}}^{Circ.}\right]\right\}\cdot \left[ABCD_{Res.N}^{Circ.}\right]\\ =\left[\begin{array}{cc} A_{Tot.} & B_{Tot.}\\ C_{Tot.} & D_{Tot.} \end{array}\right]\\ S_{11}^{Circ.}=\frac{A_{Tot.}+B_{Tot.}/Z_{0}-C_{Tot.}Z_{0}-D_{Tot.}}{A_{Tot.}+B_{Tot.}/Z_{0}+C_{Tot.}Z_{0}+D_{Tot.}}\\ S_{21}^{Circ.}=\frac{2}{A_{Tot.}+B_{Tot.}/Z_{0}+C_{Tot.}Z_{0}+D_{Tot.}} \end{array}$$

The ABCD-parameter matrix of each block of the circuit is multiplied sequentially from *P_in_* to *P_out_*. S_11_ and S_21_ are functions of the elements of the finalized ABCD matrix.

### 2.2. Devising the Metamaterial Resonators in the Waveguide

The making of a thin resonator within the cross-section of the waveguide starts at the equivalent circuit modeling for the zeroth-order resonator. Unlike the previous ZOR filters or metamaterial passive devices, which were formed mainly in the longitudinal direction as the microstrip-line or CPW, the novel ZOR is proposed to be formed in the transverse directions on the WG cross-section as a novel approach. A CRLH circuit is built by considering the up, down, left and right metallic walls.

**Figure 3:**
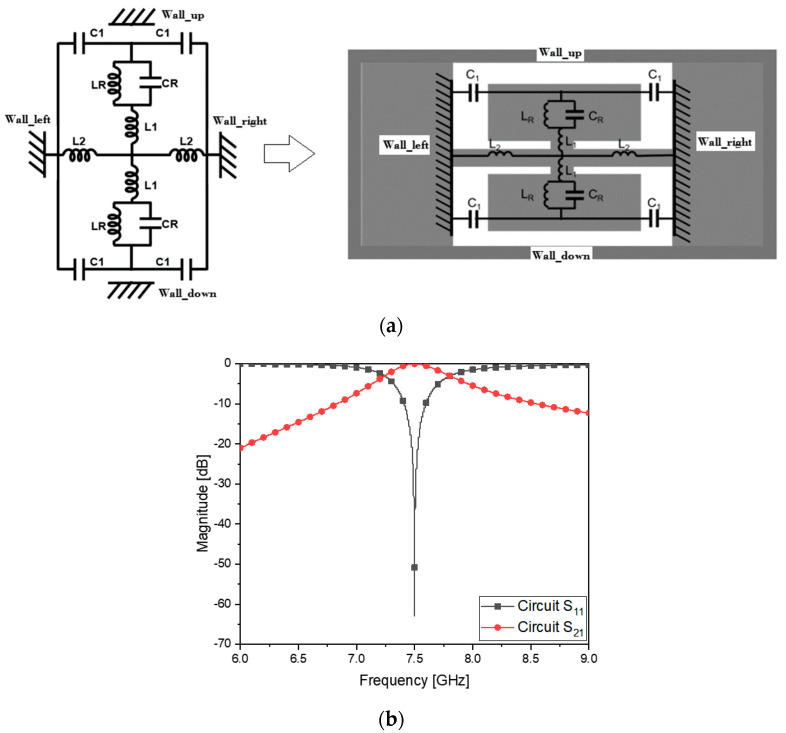

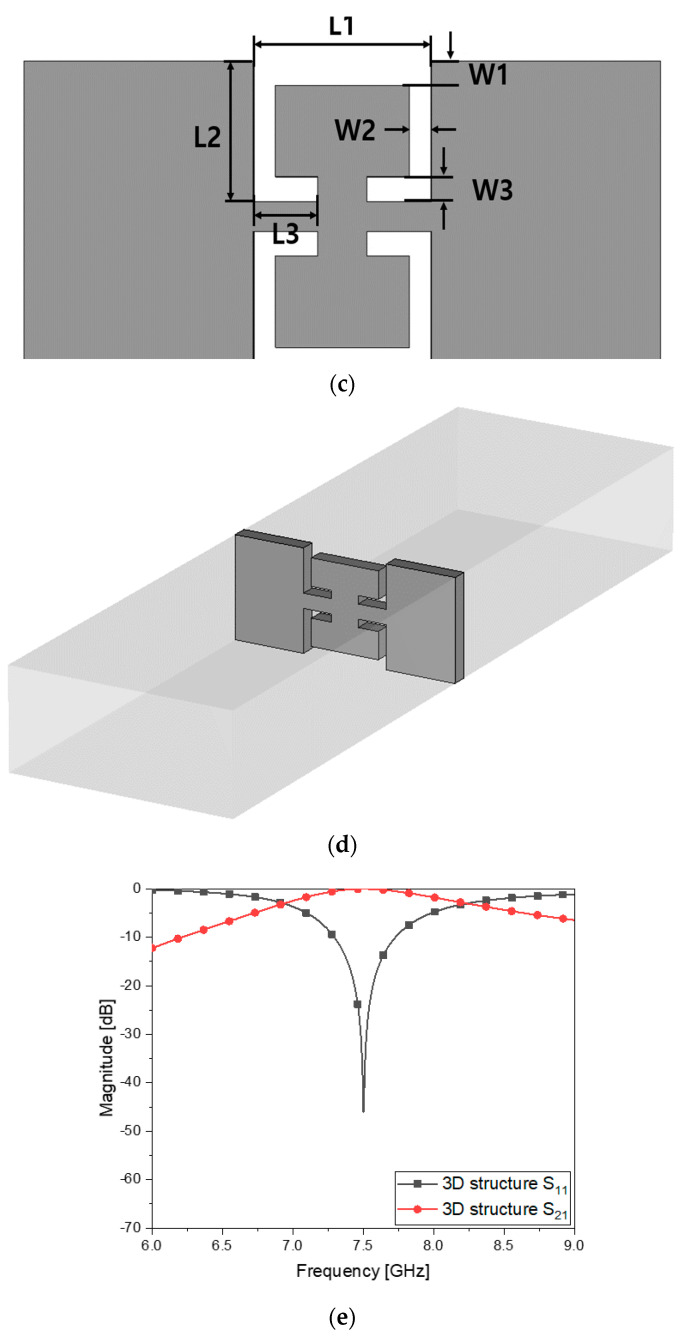
Circuit model of the CRLH resonator and move into the waveguide. (**a**) E−CRLH circuit of the resonator applied to the waveguide cross−section. (**b**) S_11_ and S_21_ of the resonator circuit. (**c**) Physical shape of the thin resonator. (**d**) The resonator is longitudinally flat. (**e**) S_11_ and S_21_ of the resonator structure.

In Figure 3a, the E-CRLH circuit is given as combination of shunt L, shunt C, series L and series C which go well with four metallic surfaces on the waveguide cross-section. C_1_ as series C from *Wall_left* to *Wall_right*, and L_2_ as shunt L from *Wall_left* to *Wall_right* are slots and short-circuited with the metal walls, spread in the transverse directions. Thinking of the vertical electric field of TE_10_-mode, a metal plate gets in the way to capture the E-field and is divided into the upper and lower patches modeled as L_R_C_R_ sub-resonators connected through a strip equivalent to L_1_. Combining the electrical attributes of the elements, for the purpose of the metamaterial resonance at the center frequency, the circuit values are obtained as follows and they are written in Table 3.

Using the values, S_21_ and S_11_ in the plot of Figure 3b show the resonance as planned.

As mentioned, the geometrical information of the slots, short-circuiting lines, metal patches and their connecting strip in Figure 3c,d is figured out by electromagnetically simulating the structure in a full-wave analysis program, on the basis of C_1_, L_2_, L_R_C_R_ and L_1_ given in Table 3 to get the same frequency response as Figure 3b. As a result, S_21_ and S_11_ of the flat WG metamaterial resonator of the physical dimensions written in Table 4 are obtained as Figure 3e agreeing with Figure 3b. Using the frequency response and electromagnetic simulation, the ZOR as the metamaterial characteristics is verifiable.

**Figure 4:**
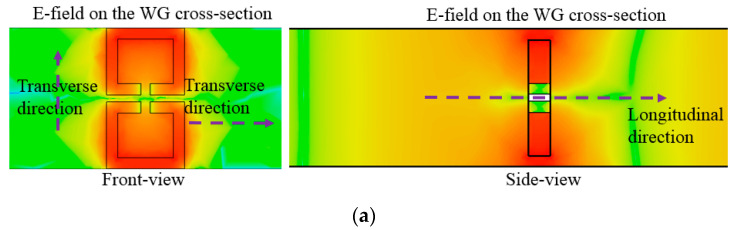

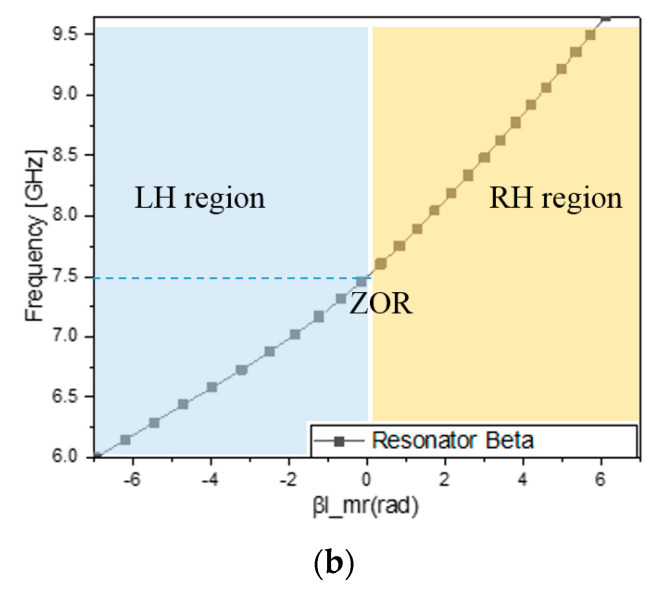
Observing the ZOR of the resonator. (**a**) E−field resonant and confined to a thin plane. (**b**) Dispersion diagram.

In the area of the conventional passive components, they resonate at the half-wave long TX-line segment, and when becoming much shorter than the half-wavelength, they are not resonant but evanescent. The field is strongly resonant on the structure far shorter than the half-wavelength as in Figure 4a. This field is observed at the target frequency. Especially in the side-view, there are two vertical bars whose gap is roughly the half-wavelength, and the thickness of the proposed resonator is a small fraction of quarter-wavelength. The electromagnetic wave entering the leftmost side (input port) propagates in the longitudinal direction, which is not blocked by this thin structure. Not as the evanescent mode, this propagation mode is consistent with the beta equal to zero occurring at the same frequency in reference to the dispersion diagram. As the relationship between beta (propagation constant) and frequency, the non-linear curve is formed from the LH (left-handed) region of negative beta through the ZOR point to the RH (right-handed) region of positive beta. For the conventional WG bandpass filters, cavities are designed as the half-wave long waveguide sections for the resonance of interest and cascaded through irises for inter-resonator coupling. Generally, irises as H-plane or E-plane steps are not resonant but have reactance. On the contrary, the proposed resonator as the sub-wavelength structure passes the RF signal on at a specific frequency. To generate a certain bandwidth as the passband and roll-off outside the band-edges, resonators must be coupled to control the amount of electromagnetic fields transmitted to the next resonator. This mechanism increases the order of the filter and the steepness of the skirt.

### 2.3. Formation of the Passband by Cascading the ZORs with Coupling Elements

The resonators are placed in series through coupling elements as quantified in Figure 2 and Table 2. The coupling elements are denoted as *length_i_* for the eighth order of filtering. Prior to the high order filter, the second-order BPF is built to see the basic characteristics of the coupling element suggested together with the ZORs.

**Figure 5:**
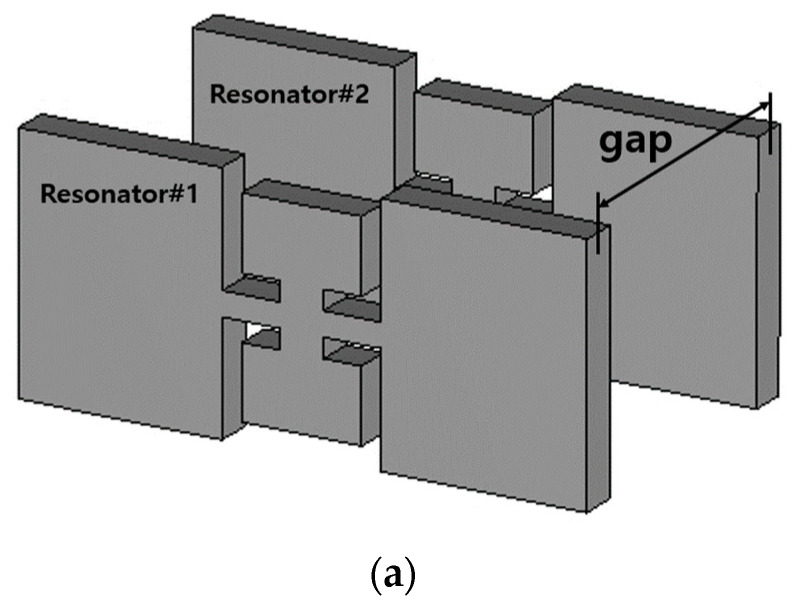

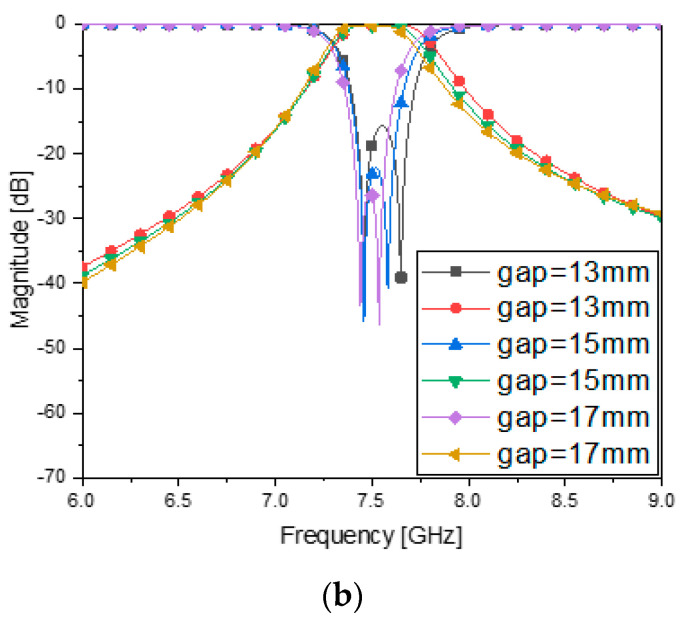
Combining two ZORs through the gap for coupling. (**a**) Structure. (**b**) S_11_ and S_21_ vs. *gap*.

Figure 5a is drawn with the geometrical parameters in Table 5. S_21_ in Figure 5b reveals the slope of the skirt has been made rapid compared to the 1-pole case in Figure 3b. The gap length of the coupling element between the resonators is obtained by finding the value which generates the desirable S_11_ and S_21_ performances, as it is varied as in Figure 5b. The gap length of 15 mm is proper for the second-order case because of the impedance matching as S_11_ becomes worse, and the bandwidth increases for gap length 13 mm and the bandwidth decreases for gap length 17 mm. Now the attenuation level in the stopband is around 10 dB. To have higher attenuation at the stopband, the structure is extended to the eighth-order filter.

**Figure 6:**
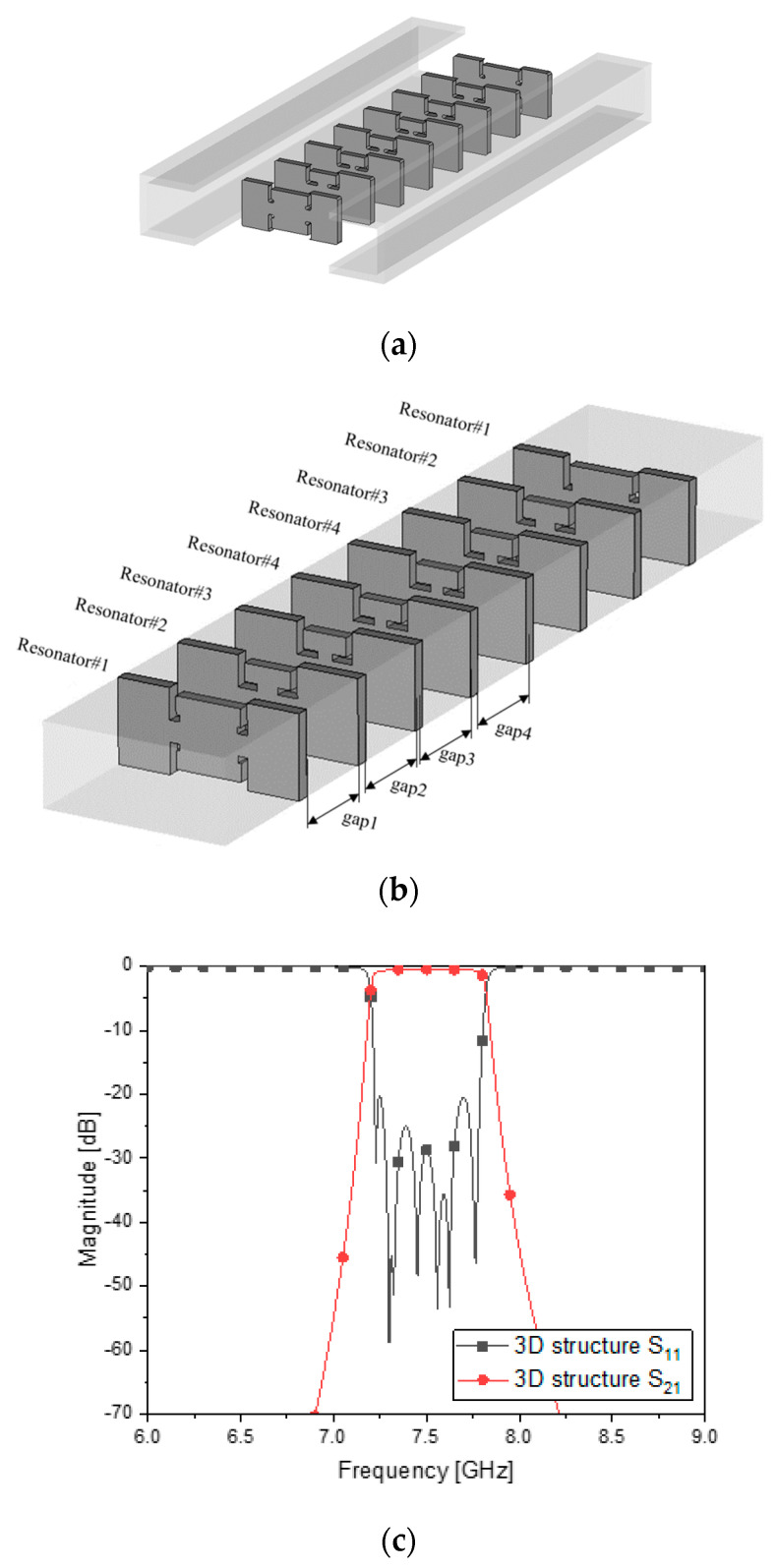

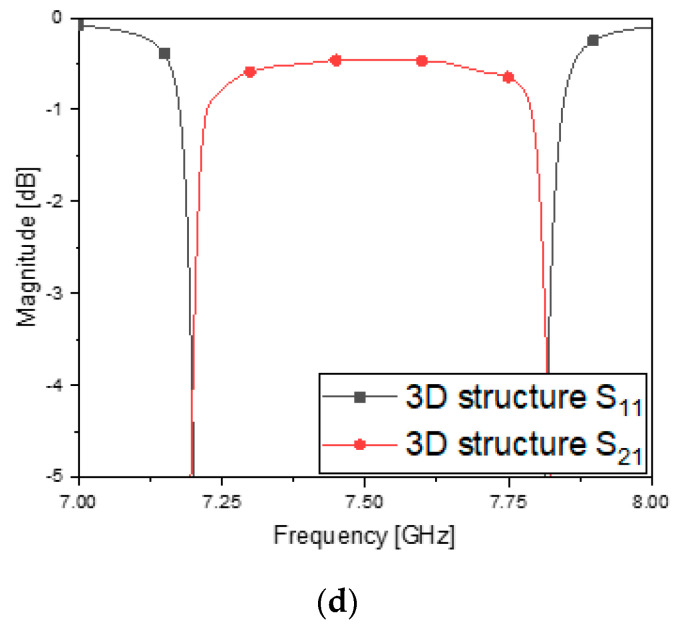
Combining eight ZORs through the gaps for coupling. (**a**) and (**b**), structure. (**c**) S_11_ and S_21_. (**d**) S_11_ and S_21_ zoomed−in.

Beyond the 2-pole BPF, for assuring a high level of noise suppression at the stopband, 8 ZORs are put in order, and adjacent resonators are coupled through *length_ij_*. Figure 6a is an open structure before assembly and Figure 6b is the complete shape. The geometric values in Table 5 change to those in Table 6 due to changing the 1 resonator as a narrow band to form the required passband by cascading eight resonators through coupling sections. The coupling required by adjacent resonators causes a change in the phase and geometric values. Giving the values to the variables as in Table 6, the full-wave EM simulation provides the designer with the frequency response of Figure 6c. Excellent impedance matching is seen through S_11_ of −20 dB and the insertion loss of S_21_ of −0.9 dB in the passband. This is also checked with Figure 6d as the magnified version. Among other things, the attenuation has been improved by a large margin with the steeper skirt. It is 40 dB.

## 3. Fabrication of the WG ZOR BPF and Test of the Prototype

The designed waveguide filter is fabricated and measured to validate the proposed method and geometry. Since the CNC milling technique is conducted for fabrication, taking into account that the end-mill tip cannot realize the aforementioned shapes of the cross-sections of the thin resonators 100%. The round corners appear instead of the sharp right-angle ones. Though the design has been done with the unit of 10 μm as a coarse approach to ease the mechanical tolerance, the round corners are inevitable. The secondary procedure of design is done to keep the function of the WG BPF satisfactory, as in Figure 6. This leads to the modified values for the geometrical parameters and the values are seen in Table 7.

**Figure 7:**
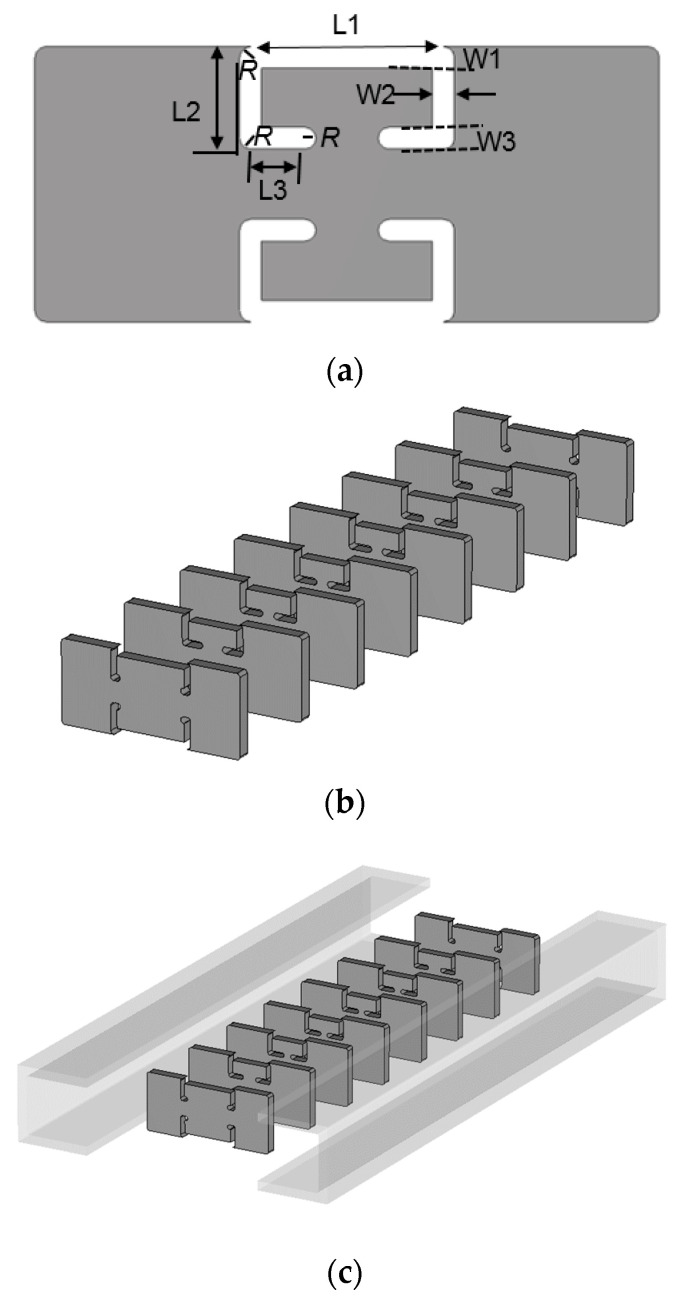

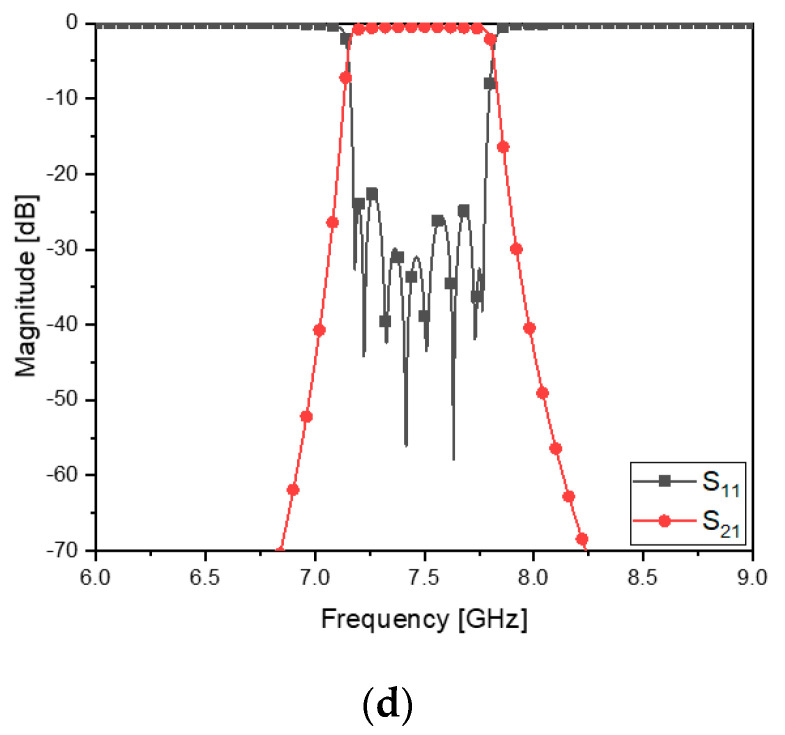
Realistic geometry of the eighth−order WG ZOR BPF. (**a**) Front-view of the thin resonator with the round corners. (**b**) and (**c**), 3D structure. (**d**) S_11_ and S_21_.

Despite the geometrical change in the front view of the flat metamaterial resonators, it is necessary to keep the frequency response compliant with the specifications. The corners become round *R* and weaken the slot capacitance and the inductance on the edges of the metal patches and their intermediate strip. The rounded corner leads to lower capacitance due to a wider gap and lower inductance due to a shorter current path. This means at a microwave frequency band it degrades the initial performance of the BPF. In the realistic case, Figure 3c and Figure 6b are rendered as Figure 7a–c. Some of the physical dimensions have to be fine to be in the unit of 100 μm; there is no more being coarse in order to achieve the required frequency response. Setting up the structure in the electromagnetic analysis software with the values for the geometrical parameters, the transmission and reflection coefficients are obtained as in Figure 7d,e, which meets the design requirement. This is very different from cavity filters presented by [15,16,17] in terms of shape and length, and is physically realized as follows.

**Figure 8:**
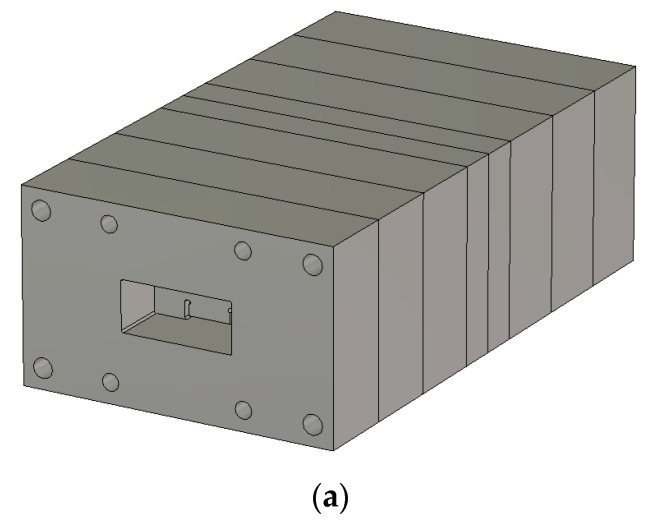

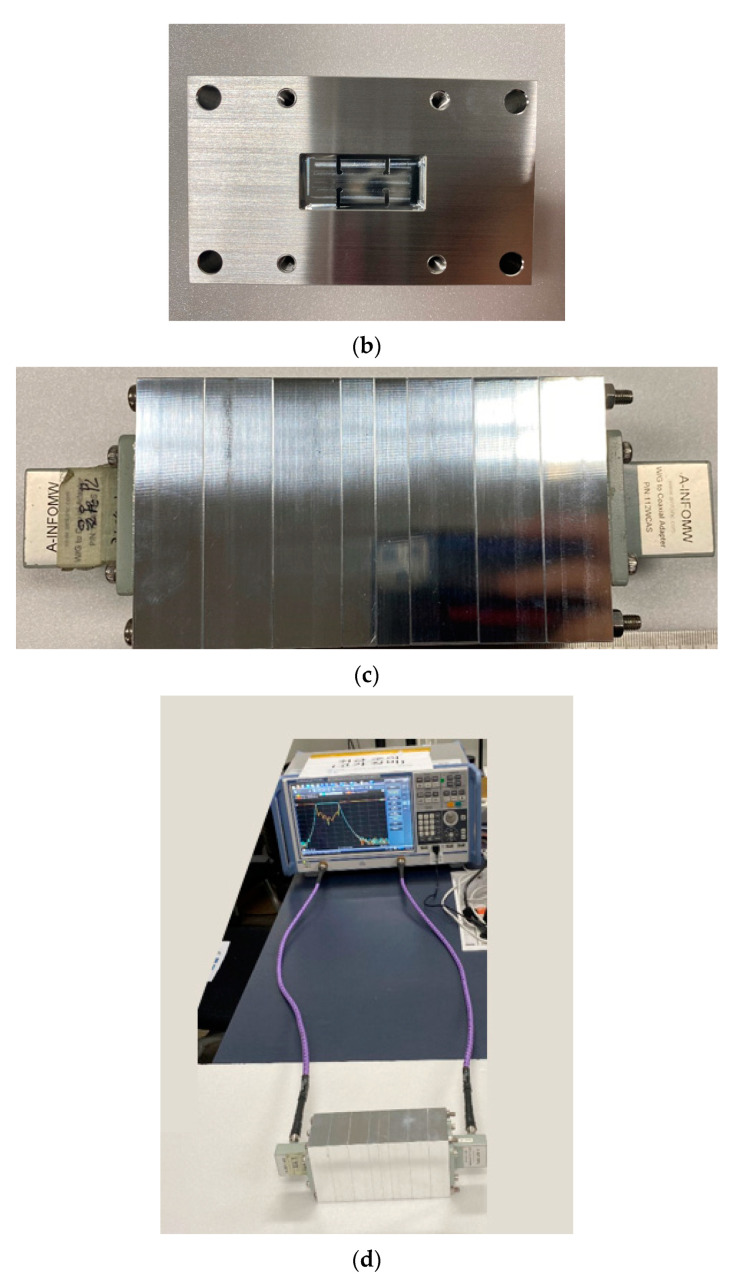

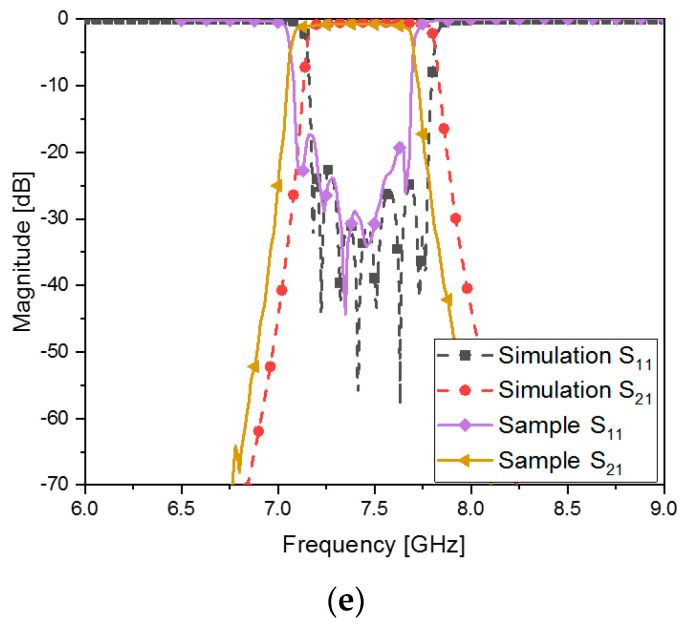
Prototyped eighth−order WG ZOR BPF via the CNC milling process. (**a**) Bird’s eye−view of the thin resonator with the round corners embedded in the flange body. (**b**) Front−view of the manufactured filter. (**c**) Top−view of the manufactured filter. (**d**) The device under test having the adaptors matched with the flanges. (**e**) S_11_ and S_21_.

The aluminum ingot is carved into WG metamaterial resonators and coupling sections in the milling process as in Figure 7a and they are pieced together to the eight-pole BPF as in Figure 7b,c. The original structure becomes a little longer and wider because it has parts for mechanical assembly with bolts and a flange body for WG port connecting. However, the size of the core has the length of 3.5 $\lambda_{g}$ for the eighth-order filtering. With this harness, the WG BPF is tested to see the frequency response. This experiment is conducted as in Figure 8d, which produces S_11_, and S_21_ is as in Figure 8e. The insertion loss and reflection coefficients are about −0.9 dB and −19 dB in the passband. The roll-off this manufactured metamaterial filter makes is satisfactory with attenuation of almost −40 dB. There occurs a discrepancy between the simulated and measured results that the frequency is shifted downward a bit. It is inferred that the error in R (round) formed by the end-mill affects the inductive part which causes the frequency shift in the first place, and secondly, connected pieces in the longitudinal direction do not tightly contact each other, making a tiny gap between metal rims with rotational misalignment. This proposed geometry as a guided component is applied to an antenna system as the electromagnetic radiation problem.

**Figure 9:**
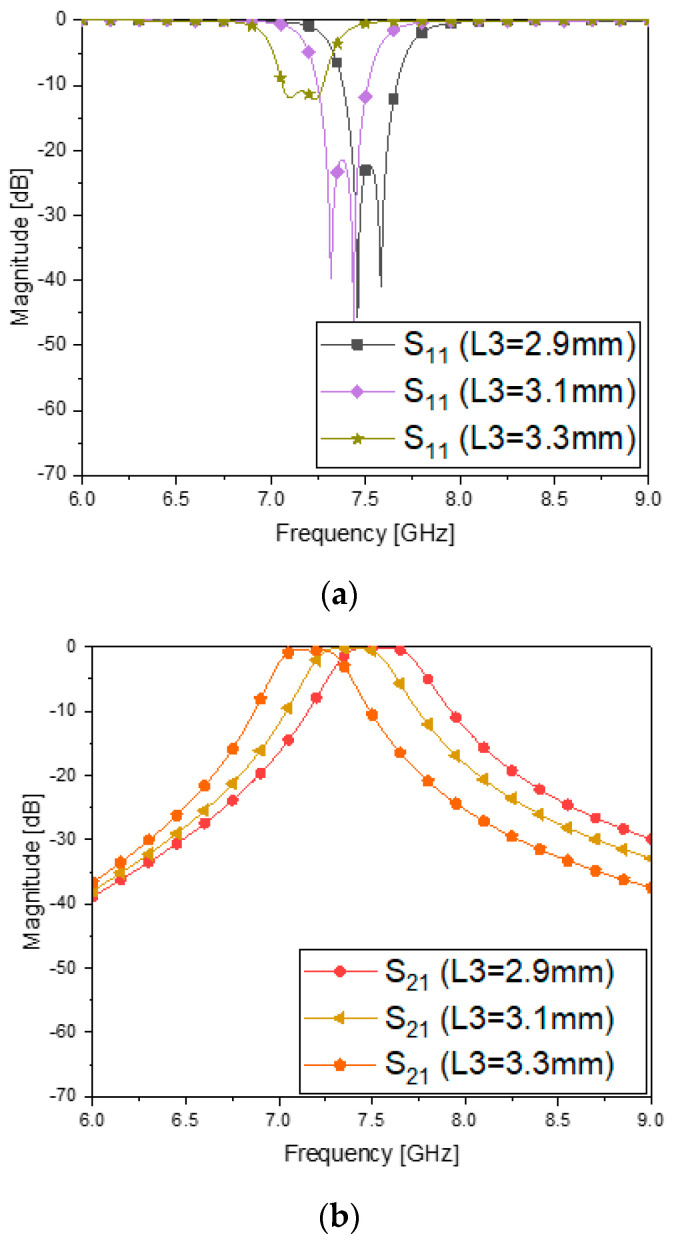

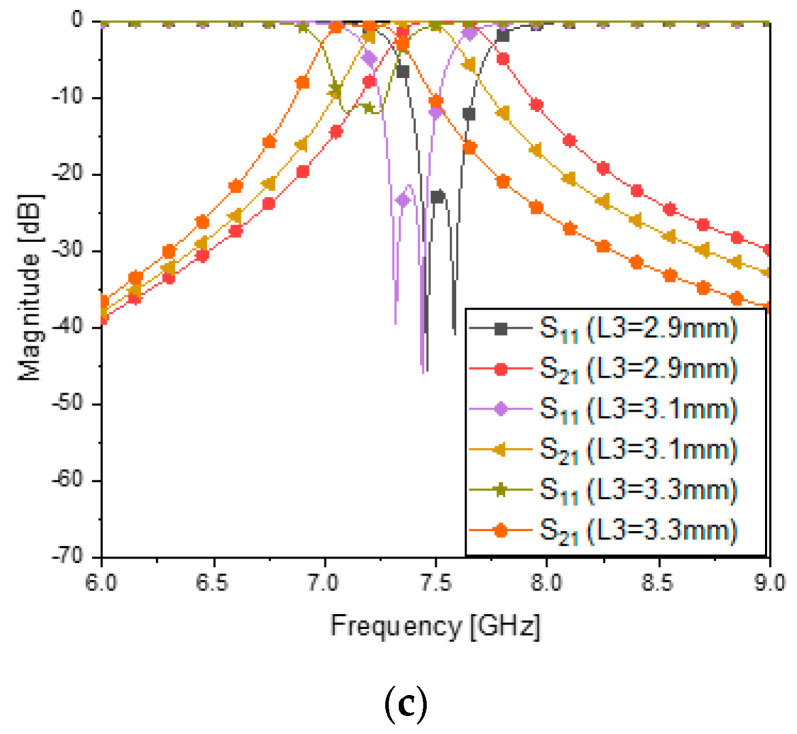
Parametric sweep to know what possibly causes the frequency shift of the BPF of the second order as an example. (**a**) S_11_ vs. variation in L3. (**b**) S_21_ vs. variation in L3. (**c**) S_11_ and S_21_ vs. variation in L3.

Figure 9 provides variation in L3 generated by the error of the rounded corner, which is critical to the change in the frequency response. For a quick simulation, a second-order case of the proposed filter is dealt with. L3 varied from 2.9 mm to 3.3 mm, mainly causing the frequency shift in S_11_, S_21_, S_11_ and S_21_ as in Figure 9a–c, respectively.

The characteristics of the proposed filter and reference BPF structures are compared as in Table 8. Most of all, the proposed filter has the shortest resonator as the WG metamaterial, which results in a good insertion loss from the complete structure at the length of 3.5 $\lambda_{g}$ for a relatively low frequency, while [5,7] have lengths of around 4.7 $\lambda_{g}$ and 8.5 $\lambda_{g}$, each for a relatively high frequency. If the proposed method is applied to 11 GHz, the total length is expected to be 3.5 $\lambda_{g}$, which is shorter than [5] according to a quick estimation. This work and refs. [5,7] are high-order filters, giving high levels of attenuation in the stopbands, but [17,18,19] take four cavities, showing poor noise-suppression effects. If [15,16,17] are elongated to high-order filters, the lengths and insertion loss will be larger. Because the operation frequency of this work is much lower than others’ and has to use a WR-112 cross section as the largest, the 7.5 GHz filter might be the heaviest from Table 1 when the same order is assumed for all the compared cases. Thus, the total length must be as small as possible, enabled by the metamaterial resonators. The proposed filter is compared with the non-metamaterial filter in terms of size and function.

## 4. Filter-Integrated Antenna Realized and Tested to Observe Channel Selectivity

The wireless communication system has transmitting (TX) and receiving (RX) antennas. Likewise, the satellite transponder has two antennas that are tested to see the RF-signal transfer and its reception between the opposite sides. The TX antenna is directly connected to the signal generator, which is practiced on a lot for checking the antenna-only function. Horn antennas are adopted for satellite wireless equipment most of the time, and because they have the characteristics of wide bands, frequency channels having several hundred megahertz of bandwidths are not defined for them. As channels are important in any communication, the roles of bandpass filters are highly in demand. Therefore, the proposed bandpass filter is inserted into the TX antenna system as below.

**Figure 10:**
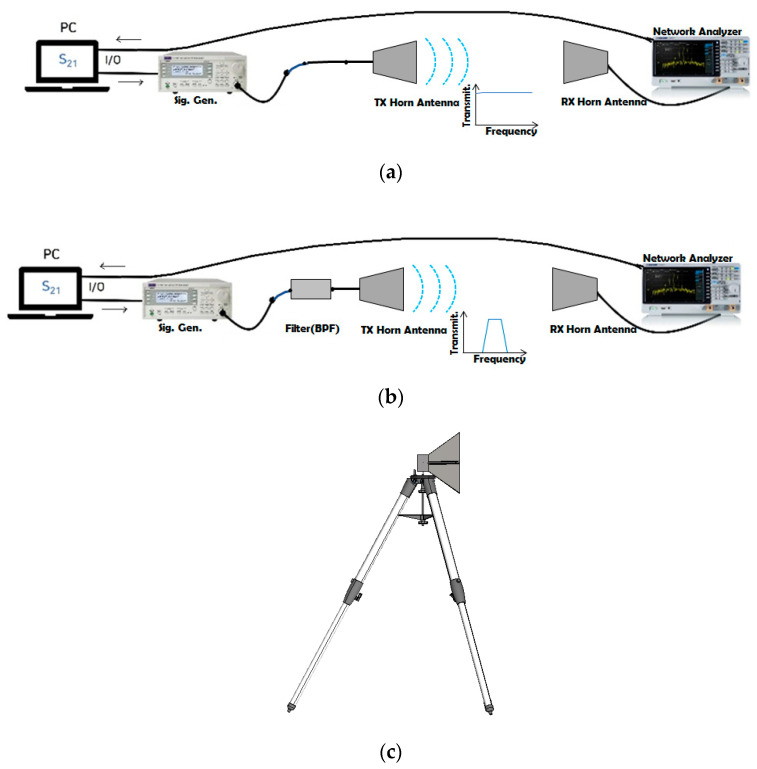

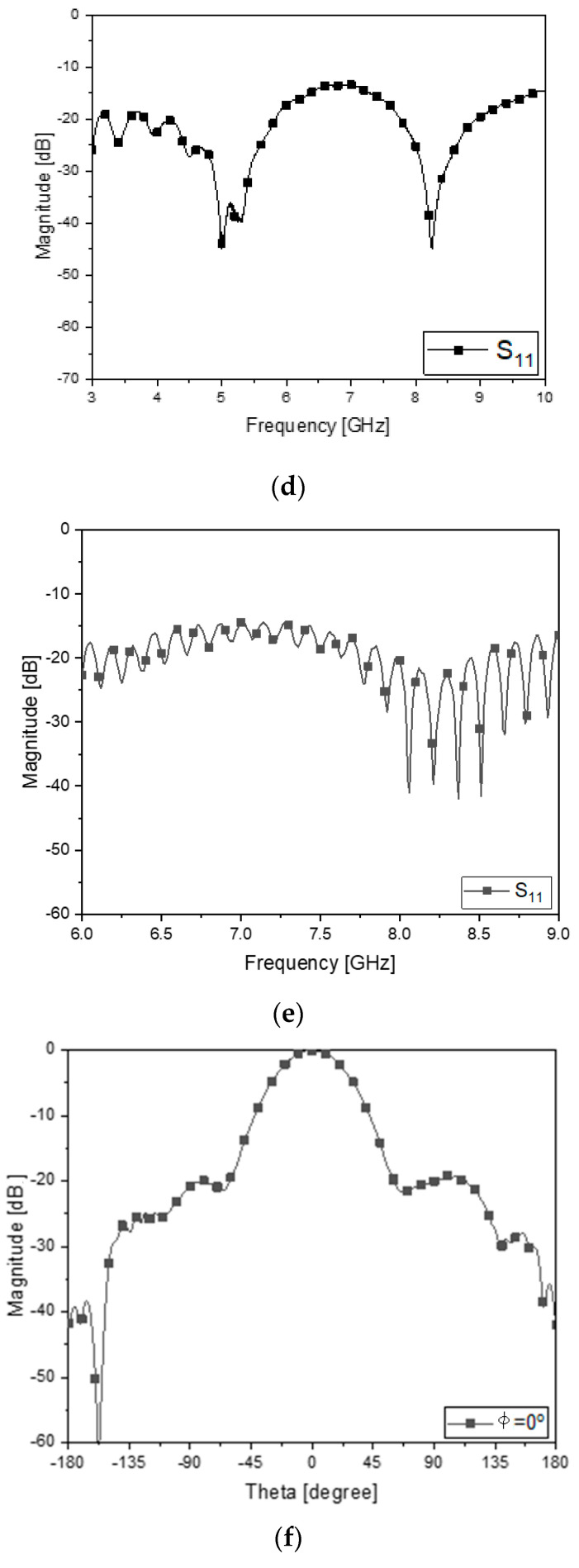

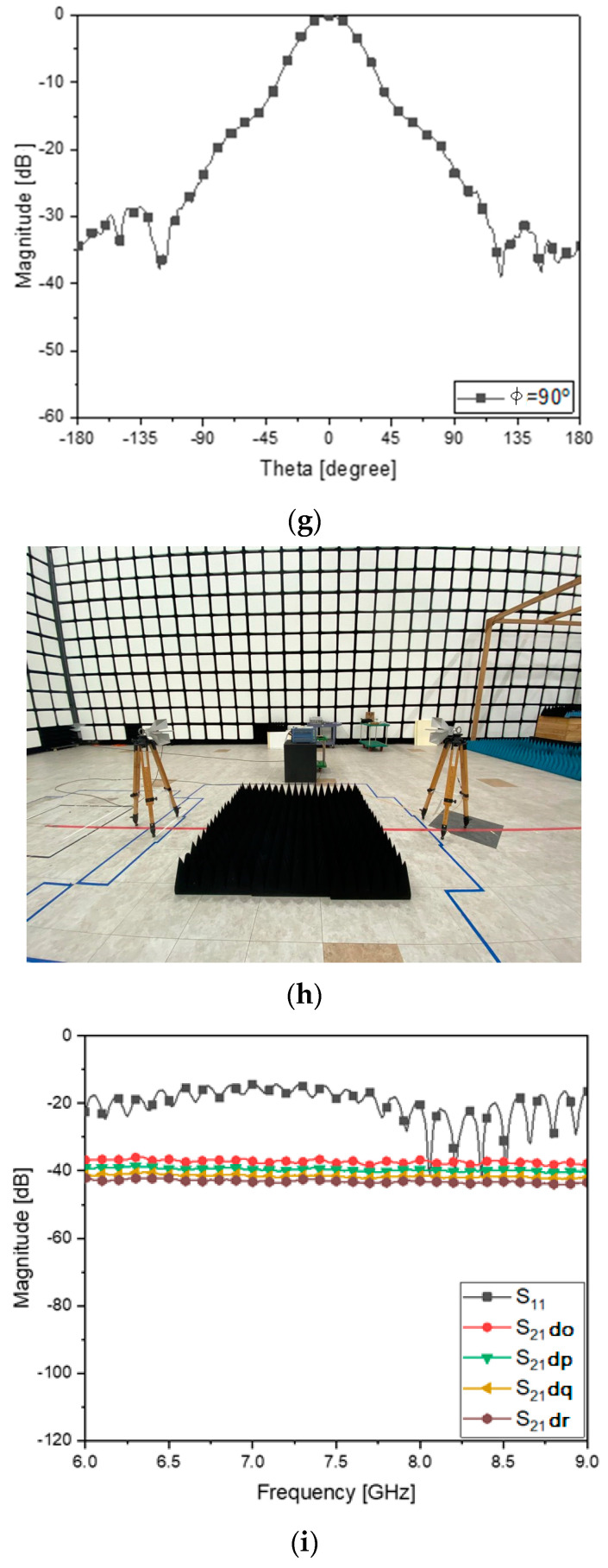
RF−link tests of the horn antennas without and with the BPF. (**a**) Signal transfer between the TX and RX horn antennas. (**b**) Case of the TX as the filtenna. (**c**) Horn antenna standing on either of the TX and RX sides. (**d**,**e**) S_11_ of the horn antenna. (**f**) Far−field pattern on the Φ = 0° plane for the horn antenna. (**g**) Far−field pattern on the Φ = 90° plane for the horn antenna. (**h**) Over−the−air test in the anechoic chamber. (**i**) S_11_ and S_21_ when the setup is without the BPF with the antenna position adjusted back and forth in a small scale such as 1 cm to 4 cm.

Figure 10a is the scheme of the ordinary RF-link test setup, while Figure 10b depicts how the TX horn antenna is fed through the BPF from the signal generator. The shape of the horn antenna given in Figure 10c is commonly used for wireless connectivity observation. It is R&S^®^HF907 made by Rohde-Schwarz. Figure 10d–g show its representative characteristics, in other words, S_11_, Φ = 0° plane beam-pattern and Φ = 90° plane beam-pattern. These beam-patterns as the field-strength measured on the angular variation are side-data, and for the RF-link investigation, the RF transmission between the TX and RX sides on a straight line as in Figure 10h expresses the field-strength. Like Figure 10d, S_11_ is below −10 dB over a very broad band as in Figure 10i. S_21_ as the RF transmission between the TX and RX antennas was measured over the distance ranging from 3 m (denoted as *do*) to 6 m (as *dr*), and has the feature of a broad band. Signals can be contaminated with noise and interference in that situation. Channel selectivity necessary for wireless communication is made possible by the following method foreseen in Figure 10b.

**Figure 11:**
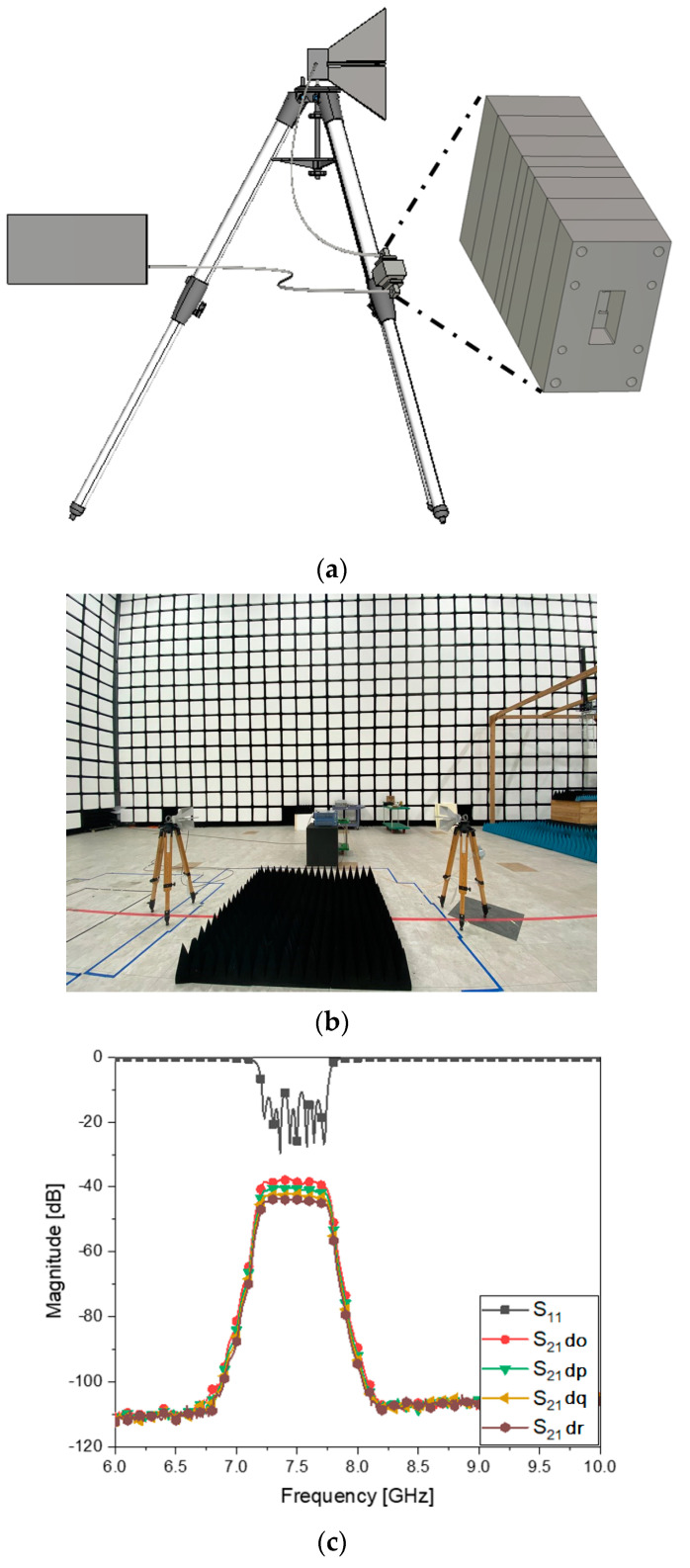
RF−link and channel selectivity test. (**a**) Case of the TX as the filtenna that comprises the BPF and the antenna. (**b**) Over−the−air test. (**c**) S_11_ and S_21_ when the setup places the BPF in the feed for the TX antenna.

Figure 11a includes a gray rectangle denoting the proposed metamaterial BPF as part of the feed of the TX horn antenna. The real experiment was conducted as in Figure 11b. The field strength from the filter-incorporated antenna was measured by the RX horn antenna, which was plotted as Figure 11c. Channel selectivity is accomplished as revealed by S_11_ as the input reflection coefficient of the TX horn and S_21_ as the signal transfer between both the sides. The two s-parameters have clear distinction of the passband and stopband like in Figure 7. The antenna system is enabled to select the signals as required.

**Figure 12:**
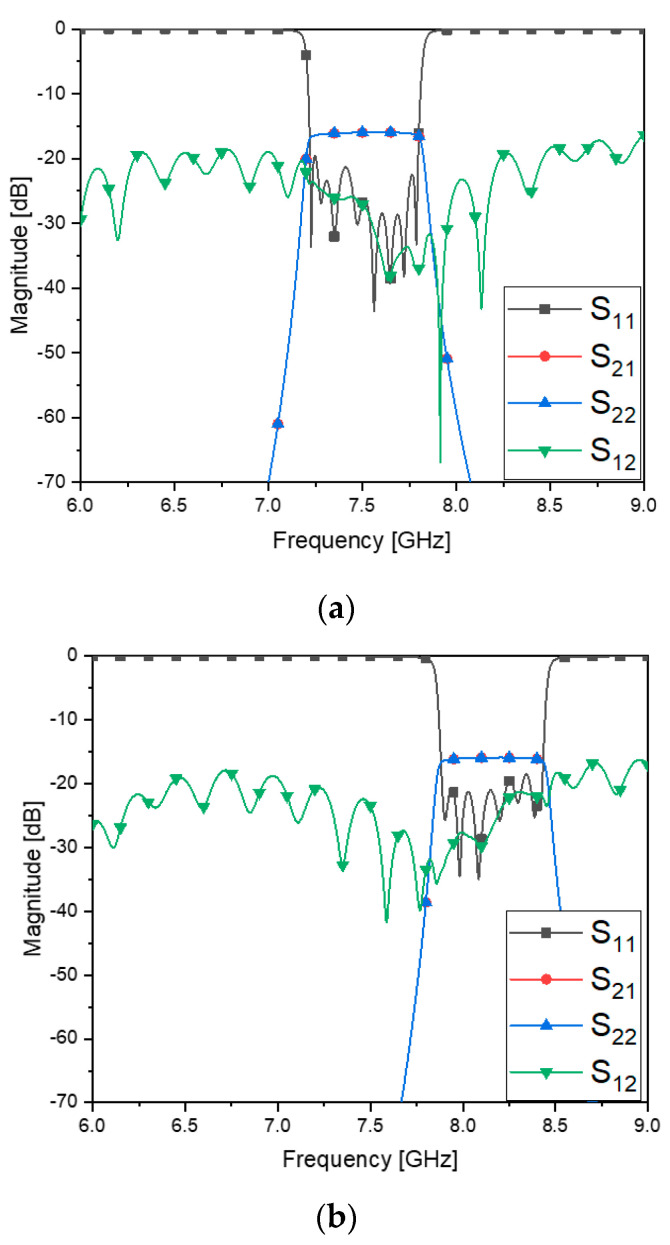
RF−link and channel selectivity test which assumes a frequency change in the bandpass filter block. (**a**) S-parameters of filter−combined antenna with the frequency of the BPF before the change. (**b**) S−parameters of filter−combined antenna with the frequency of the BPF after the change.

As an extension of the over-the-air test, since the simulation of the three-dimensional structure is time-consuming, simple numerical experiments are conducted. Figure 12a has the frequency responses of the passband of the filter on the TX-side, which is combined with the antenna. Figure 12b means the received signal has a plot presenting the frequency shift in S_21_ as the result of the frequency shift occurring to the TX side. This kind of test makes the proposed work different from other suggestions [18,19], where the over-the-air transmission is not investigated. Table 9 compares their works with this work from various view-points.

As in Table 9, the features of the latest articles showing the filter combined with an antenna and this work are dealt with in the aspects of operation frequency, bandwidths, orders of the filter, waveguide cross-section sizes and lengths. The frequency and bandwidth of [18] is relatively easy to achieve, but that of this work has the highest level of difficulty in design from the stand points of fractional bandwidth and skirt slope. The orders of filtering of [18,19] are much lower than that of this work. However, the proposed structure is the most compact in spite of the highest order of filtering. In other words, the size-reduction effect of the waveguide CRLH geometry is superior to that of the references.

## 5. Conclusions

A novel design method and geometry of the waveguide bandpass filter are suggested. Substantial size-reduction and excellent bandpass filtering functions are made possible by coming up with the waveguide CRLH resonator which leads to a very thin structure much shorter than the half-wavelength for the conventional cavities. The ZOR phenomenon is generated with the transverse geometrical parameters of the waveguide cross-section, unlike other metamaterials utilizing longitudinal line segments. The ZOR as the thin waveguide part does not block the incoming RF signal but passes it to the next ZOR. By cascading the ZORs through transmission-sections as the coupling elements, the passband becomes distinct with the steeper skirt in the stopband. An eighth-order ZOR BPF is designed and simulated, moving to the step of fabrication. It is manufactured into the aluminum waveguide filter. The prototyped BPF is measured and compared with the simulated result. As for the passband, insertion loss and reflection coefficient are around ≤1 dB and ≤−15 dB from simulation to measurement. The attenuation of ≥40 dB at 7 GHz and 8 GHz is achieved as desired in the specifications. The length of the WG ZOR BPF is 3.5 $\lambda_{g}\;$ for the eighth-pole, but the conventional one has 5 $\lambda_{g}$ even for the seven-pole case. Furthermore, the BPF was combined with the horn antenna in order to provide it with channel selectivity. The broad-band horn antenna is made possible to select the signals through the passband. The frequency responses are acceptable for the use of a satellite transponder. The proposed filters make the LEO- and scientific satellites much lighter with the weight of 54 g, which is greatly reduced from the conventional WG filters.

## Figures and Tables

**Table 1:** Specifications the design will meet.

Item	Value
Insertion Loss	≤1 dB
Center Frequency	7.5 GHz
Bandwidth	500 MHz
Reflection coefficient	≤−15 dB
Out of Band Rejection (or Skirt)	≤−40 dB(fc ± 500 MHz)

**Table 2:** Calculated unknowns of Figure 1 and Figure 2 for the required BPF.

Variable	Value	Variable	Value
$L_{P1}$	0.998 nH	$L_{P2}$	0.516 nH
$L_{P3}$	0.417 nH	$L_{P4}$	0.408 nH
$C_{P1}$	0.442 pF	$C_{P2}$	0.859 pF
$C_{P3}$	1.064 pF	$C_{P4}$	1.088 pF
*length1*	20 mm	*length2*	15.6 mm
*length3*	16.6 mm	*length4*	16.5 mm
*length5*	16.6 mm		

**Table 3:** The values of the circuit elements of the E−CRLH resonator.

Variable	Value	Variable	Value
*L_R_*	1.6 nH	*C_R_*	2.27 pF
*L1*	0.586 nH	*C1*	0.626 pF
*L2*	1.51 nH		

**Table 4:** The values of the physical dimensions of the E-CRLH resonator.

Variable	Value [mm]	Variable	Value [mm]
*L1*	8.8	*W1*	1
*L2*	4.7	*W2*	1
*L3*	3.4	*W3*	1

**Table 5:** The values of the physical dimensions of the second-order filter.

Variable	Value [mm]	Variable	Value [mm]
*L1*	8	*W2*	1
*L2*	5.7	*W3*	1
*L3*	2.9	*gap*	15
*W1*	1		

**Table 6:** The values of the physical dimensions of the eighth-order filter.

**Resonator#1**			
**Variable**	**Value [mm]**	**Variable**	**Value [mm]**
*L1*	12.2	*W1*	1
*L2*	4.7	*W2*	1
*L3*	1.5	*W3*	1
**Resonator#2**			
**Variable**	**Value [mm]**	**Variable**	**Value [mm]**
*L1*	9.1	*W1*	1
*L2*	4.7	*W2*	1
*L3*	3.1	*W3*	1
**Resonator#3**			
**Variable**	**Value [mm]**	**Variable**	**Value [mm]**
*L1*	8.8	*W1*	1
*L2*	4.8	*W2*	1
*L3*	3.3	*W3*	1
**Resonator#4**			
**Variable**	**Value [mm]**	**Variable**	**Value [mm]**
*L1*	8.8	*W1*	1
*L2*	4.7	*W2*	1
*L3*	3.4	*W3*	1
**Gap**			
**Variable**	**Value [mm]**	**Variable**	**Value [mm]**
*gap1*	15.8	*gap3*	15.2
*gap2*	15.5	*gap4*	15.6

**Table 7:** The values of the physical dimensions of the eighth-order filter, considering fabrication.

**Resonator#1**			
**Variable**	**Value [mm]**	**Variable**	**Value [mm]**
*L1*	12.77	*W1*	1
*L2*	4.7	*W2*	1
*L3*	1.74	*W3*	1
**Resonator#2**			
**Variable**	**Value [mm]**	**Variable**	**Value [mm]**
*L1*	9.8	*W1*	1
*L2*	4.7	*W2*	1
*L3*	3.48	*W3*	1
**Resonator#3**			
**Variable**	**Value [mm]**	**Variable**	**Value [mm]**
*L1*	9.15	*W1*	1
*L2*	4.7	*W2*	1
*L3*	3.84	*W3*	1
**Resonator#4**			
**Variable**	**Value [mm]**	**Variable**	**Value [mm]**
*L1*	9.1	*W1*	1
*L2*	4.7	*W2*	1
*L3*	3.86	*W3*	1
**Gap**			
**Variable**	**Value [mm]**	**Variable**	**Value [mm]**
*gap1*	15.4	*gap3*	15.3
*gap2*	16.8	*gap4*	14.9

**Table 8:** Comparing the characteristics of the proposed filter and others’ filters.

	$f_{0}$ (GHz)	Insertion Loss (dB)	Attenuation (dB)	WG Flange	$\mathrm{Resonator}\;\mathrm{Length}\;(\lambda_{g})$	Meta- Material
[5]	11	<1	55	WR-90	0.68	X
[7]	11	<1	50	WR-90	0.51	X
[15]	30	2	4.6	WR-28	0.57	X
[16]	9.45	0.08	>20	WR-90	0.51	X
[17]	8.175	0.35	7.9	WR-112	0.41	X
This work	7.5	0.9	40	WR-112	0.05	O

**Table 9:** Comparing the characteristics of the proposed filter and others’ filters with antennas.

	$f_{0}$ (GHz)	Bandwidth (GHz)	Order of the Filter	WG Flange	$\mathrm{Resonator}\;\mathrm{Length}\;(\lambda_{g})$	$\mathrm{Filter}\;\mathrm{Length}\;(\lambda_{g})$
[18]	32	2 GHz	4	WR-28	0.59	5.18
[19]	10	0.2 GHz	4	WR-90	0.61	2.85
This work	7.5	0.5 GHz	8	WR-112	0.05	3.5

## Data Availability

Not applicable.
